# Supplementary figures and images for: Infrared spectroscopic characterization of sesamin, a dietary lignan natural product
Source: PLoS One. 2024 Oct 11;19(10):e0296541. doi: 10.1371/journal.pone.0296541 (PMC11469502; doi:10.1371/journal.pone.0296541)

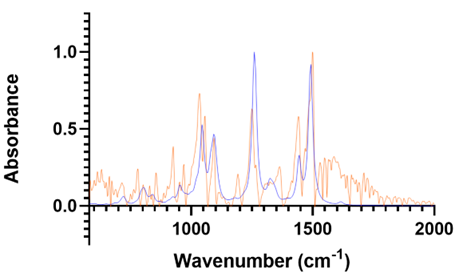

Supplement: S1 Fig — Experimental spectrum (orange); in vacuo isomer 8 (blue). (TIF) [file pone.0296541.s001.tif]

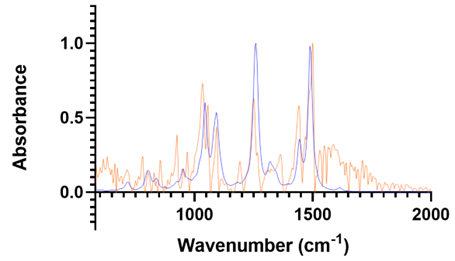

Supplement: S2 Fig — Experimental spectrum (orange); in vacuo isomer 7 (blue). (TIF) [file pone.0296541.s002.tif]

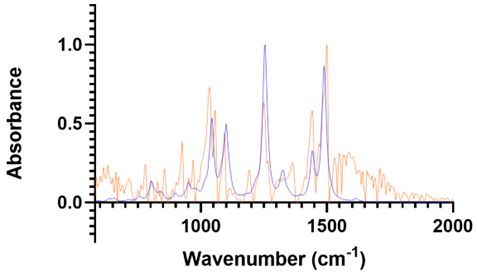

Supplement: S3 Fig — Experimental spectrum (orange); in vacuo isomer 6 (blue). (TIF) [file pone.0296541.s003.tif]

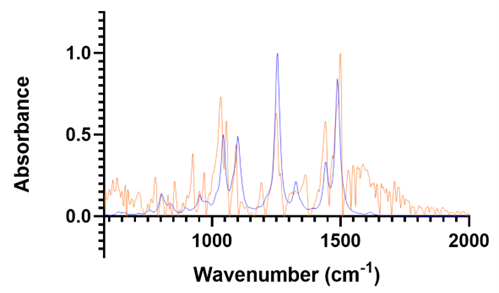

Supplement: S4 Fig — Experimental spectrum (orange); in vacuo isomer 5 (blue). (TIF) [file pone.0296541.s004.tif]

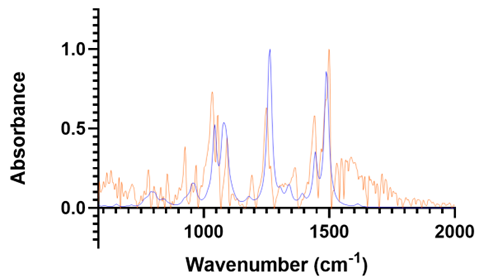

Supplement: S5 Fig — Experimental spectrum (orange); in vacuo isomer 10 (blue). (TIF) [file pone.0296541.s005.tif]

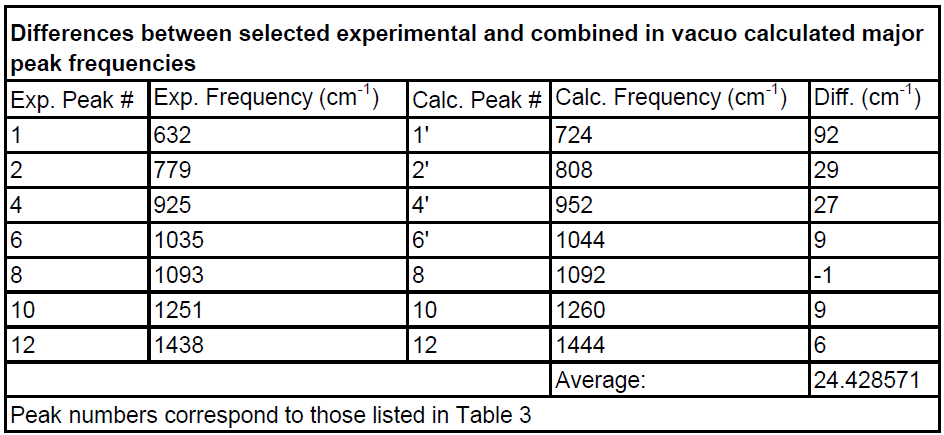

Supplement: S1 Table — (TIF) [file pone.0296541.s015.tif]
