# Supplementary material for: Infrared spectroscopic characterization of sesamin, a dietary lignan natural product
Source: PLoS One. 2024 Oct 11;19(10):e0296541. doi: 10.1371/journal.pone.0296541 (PMC11469502; doi:10.1371/journal.pone.0296541)
Supplement: Attachment — Submitted filename: Response to Reviewers.docx [file pone.0296541.s016.docx]

Dear Dr. O’Reilly,

This document lists our answers to your and the reviewers’ comments and questions.

We are grateful for these comments and reviews that have helped us improve our manuscript and its clarity. We are answering the individual reviewers’ comments separately below:

Reviewer #1: *The authors have attempted to use IR to elucidate the different stereoisomers of sesamin. It should be noted that IR alone may not be sufficient to definitively identify stereoisomers. While IR spectroscopy provides valuable information about functional groups and molecular vibrations, it might not distinguish between stereoisomers that have the same atoms and functional groups but differ in their three-dimensional arrangement. NMR spectroscopy, specifically using chiral solvents or chiral shift reagents, can also provide information about the configuration of a chiral center by observing the splitting patterns or chemical shifts of surrounding atoms. Chromatographic techniques with chiral stationary phases and polarimetry are also used for chiral analysis, although they might not directly determine the configuration of a chiral carbon but can help identify enantiomers. Thus, it is better to do some NMR calculation in these molecules instead. The basis set also that they used is too small to distinguish such changes. As such, this paper is not suitable for publication in its present state.*

We do appreciate the reviewer’s points, as well as the additional references. The 2021 paper by Majdalawieh, *et al*, is already cited by us (Ref #4) in the second sentence of our manuscript, where we introduce sesamin and the (limited) vibrational work available. The 2005 paper by Hsieh, *et al*, is indeed a paper we did not initially cite, and we are thankful to Reviewer #1 for pointing out that work to us. That paper is interesting in that it provides mostly structural data, but data related to NMR and vibrational spectra is very limited, and essentially devoid of information. We have, however, added this paper as reference #8 to replace our former #8 citation to a generic spectral database.

On the more general point raised by the reviewer: The goal of the manuscript is not to generate and comment on a magnetic resonance spectrum of sesamin. We do agree that this infrared report is not in itself an exhaustive classification of all the vibrations that are present on all isomers in every hydration condition. And indeed, our conclusion is that it is quite difficult to assertively assign peaks to a given isomer and hydration condition. Indeed, more and more diverse experiments would be needed to achieve such a goal. However, that was not our goal, and the experimental/computational data reported in this present manuscript will at least give a global “IR fingerprint” of sesamin, in several possible states and structures.

Reviewer #2: *This manuscript investigates sesamin, a lignan in sesame seed oil, by Fourier-transform infrared spectroscopy. The study compares the experimental spectrum with quantum chemical calculations of sesamin's stereoisomers, revealing potential hydrated and non-hydrated conformers. The findings pave the way for a comprehensive understanding of its structure and potential applications in pharmacology.
The work general employs correct methods and follows the right principles.
That being said, the employed methods are largely straightforward and part of the discussion presented in the manuscript should be approached with reserve. While credit may be given to the effort, the apparent in-depth discussion of the variations in the theoretical spectra of complexes with different number of water molecules are largely speculative (or, "academic"). In vacuo calculations for such systems are still only roughly representative to a solid/hydrated sample. This should be better reflected in the manuscript to prevent misleading readers not being experts in this area.
Nonetheless, the collected results and interpretations provide useful contribution to the field and I find this work publishable, provided a minor revision is considered by the authors.*

We do appreciate both the encouraging and critical comments of the reviewer. Indeed, the goal of the paper, as indicated above in response to Reviewer #1, is not to provide a definitive, fine spectral characterization of sesamin, but to provide the first step toward such a longer-term goal. We have further clarified this in the introduction sentence starting on line 45 of the manuscript.

*1. The title and the abstract ("This work provides the first experimental near infrared spectrum of sesamin") both specifically mentions NIR region, yet NIR spectrum is not presented in this work at all. Instead, the reader is informed that NIR region is not interesting, i.e., "Wavenumbers below 500 cm-1 (and above 3500 cm-1) were not considered in the peak assignment since signal was low compared to noise.*

The reviewer is entirely correct. We have corrected the title and the body of the text to state “infrared” instead of “near-infrared”.

*2. B3LYP/6-31G* by contemporary standards should not be considered "appropriate for IR spectra calculations". But, judging from the figures shown in this work, it’s sufficient in the presented case.*

We have used EDF2/6-31G* for vibrational analysis (on page 4), not B3LYP/6-31G*. The initial calculations of the potential energy differences in the sesamin isomers listed in Table 1 were done with B3LYP, which is what we indicated in the original version of the manuscript. However, these same interaction energies were re-calculated using EDF2/6-31G* and those are the values indicated in Table 2 of the manuscript. We appreciate that our initial mention of B3LYP calculations was needlessly confusing. Since all the results given in the manuscript were obtained using the EDF2/6-31G* level of calculation, we have removed that mention of (previous) calculations at the B3LYP level.

*3. What are the symmetries of each of the considered structures? If they differed by the number of symmetry elements, then the Eq. 1 should be refined with the use of symmetry cofactors (see, e.g. DOI: 10.3390/molecules24112189).*

The structures have C1 symmetry (identity). We have hence not modified the calculations based on symmetry considerations.

*4. Molecular vibrations are 'antisymmetric' not 'asymmetric'*.

Indeed, and this has been corrected in the new version. We thank the reviewer for pointing out that error to us.

5. *Experimental FTIR spectra are of poor quality (noisy, distorted baseline with negative peaks, deformed bands), which is difficult to understand, as the measurements should be fairly straightforward*.

We assume that the reviewer is referring to the experimental spectra in our manuscript and not to IR spectra in general. We appreciate the limitations of the current experimental spectra. We now clarify in the revised manuscript that further, more refined experimental spectra may be collected.

*6. Previous works modelling IR spectra on the basis of Boltzmann-weighted spectra of co-existing forms, done using anharmonic calculations and yielding highly accurate theoretical lineshape should be cited (e.g., 10.1021/acs.jpca.6b11734, 10.1038/s41598-019-53827-6, or 10.1016/j.molliq.2014.02.028).*

We thank the reviewer for pointing out these references to us. Indeed, they are very useful, and we have incorporated them in our revisions. Two of these are briefly referenced in the manuscript’s Introduction (10.1021/acs.jpca.6b11734 and 10.1016/j.molliq.2014.02.028). In the Conclusions section, another paper suggested by the reviewer, DOI: 10.3390/molecules24112189, which uses the same Boltzmann-weighting method, is referenced and comparisons are made between its major bands data and ours. We have added a Supporting information table (S1_Table) on that point. The differences these authors report between their calculated and experimental peaks are on the same order as the frequency differences that we observe between our calculations and our experimental peaks.

We hope that this revised manuscript will be found suitable for publication, and we thank you for your editing work as well as the reviewers for their time and comments.

Jerome Baudry, Ph.D.

*Mrs. Pei-Ling Chan Chair* and Professor.
