# Supplementary material for: Infrared spectroscopic characterization of sesamin, a dietary lignan natural product
Source: PLoS One. 2024 Oct 11;19(10):e0296541. doi: 10.1371/journal.pone.0296541 (PMC11469502; doi:10.1371/journal.pone.0296541)
Supplement: Attachment — Submitted filename: Answer_to_Reviewers.docx [file pone.0296541.s017.docx]

June 3, 2024

Dear Dr. O’Reilly,

Please find our revised manuscript, we have addressed Reviewer 4’s comments about the paper reporting FTIR characterization of sesamin content in seeds. This was indeed interesting to cite for the context of our work, and we appreciate the suggestion.

We hope that this revised manuscript will be found suitable for publication, and we thank you for your editing work as well as the reviewers for their time and comments.

Jerome Baudry, Ph.D.

*Mrs. Pei-Ling Chan Chair* and Professor.
